# Walking across Wikipedia: a scale-free network model of semantic memory retrieval

**DOI:** 10.3389/fpsyg.2014.00086

**Published:** 2014-02-11

**Authors:** Graham W. Thompson, Christopher T. Kello

**Affiliations:** Cognitive and Information Sciences Department, University of California MercedMerced, CA, USA

**Keywords:** scale-free network, levy foraging, category recall, semantics

## Abstract

Semantic knowledge has been investigated using both online and offline methods. One common online method is category recall, in which members of a semantic category like “animals” are retrieved in a given period of time. The order, timing, and number of retrievals are used as assays of semantic memory processes. One common offline method is corpus analysis, in which the structure of semantic knowledge is extracted from texts using co-occurrence or encyclopedic methods. Online measures of semantic processing, as well as offline measures of semantic structure, have yielded data resembling inverse power law distributions. The aim of the present study is to investigate whether these patterns in data might be related. A semantic network model of animal knowledge is formulated on the basis of Wikipedia pages and their overlap in word probability distributions. The network is scale-free, in that node degree is related to node frequency as an inverse power law. A random walk over this network is shown to simulate a number of results from a category recall experiment, including power law-like distributions of inter-response intervals. Results are discussed in terms of theories of semantic structure and processing.

## Introduction

Semantic knowledge is a core component of language processing and other advanced cognitive functions. Words and concepts have meanings and relations to each other that correspond with our experiences in the world. A long-standing, fundamental question in psychological and cognitive sciences is how semantic knowledge is represented, organized, and searched. In terms of words representation, many approaches are based on features like animacy, size, valence, etc. (McRae et al., 2005). Other approaches focus on encyclopedic-like knowledge structures (Miller George, 1995), while still other approaches use statistical co-occurrences of words in corpora as an indirect assay of semantic relations (Dumais and Landauer, 1997).

In addition to the semantic representations of words and concepts, one can also consider how they are organized in memory. One approach is to theorize semantics as a high-dimensional feature space, where individual words and concepts are points or regions in that space (Lund and Burgess, 1996). Another approach is to theorize semantics as a network with nodes representing words and concepts, and connections among nodes representing semantic relations and associations (Collins and Loftus, 1975). Any such memory structure—whether a feature space, network, or something else—must be learned, accessed, and maintained over time. There are numerous theories on how these memories are learned and accessed (see Rogers and McClelland, 2004), but here we focus on recalling items from semantic categories, and how recall relates to the organization of semantic memory.

The task of recalling items for a semantic category like “animals” or “tools” has been used for decades to assess semantic memory (Bousfield and Sedgewick, 1944), and test for neurological dysfunction (Murphy et al., 2006). Participants typically recall as many items from long-term memory as they can within a minute or two. The number of responses serves as a standard measure of semantic fluency, and items tend to be recalled in semantic, within-category clusters. For instance, for the category “animals,” a number of farm animals might be recalled first, followed by a set of house pets, then sea creatures, and so on. This pattern of behavior suggests that semantic memory is effectively clustered with respect to this recall task, and analyses of inter-response intervals (IRIs) corroborate this hypothesis—IRIs are shorter within clusters vs. between clusters (Gruenewald and Lockhead, 1980). Moreover, recall behavior seems to approach an optimal recall rate, in that participants tend to switch to a new “patch” when IRIs increase beyond the expected mean IRI (Hills et al., 2012; but see Abbott et al., 2012).

We are interested in the clustering of semantic memory and how it is reflected in recall performance. Given the evidence for a relationship between IRIs and clustering of semantic categories, the distribution of IRIs for a recall session might provide an overall index of the statistical structure of semantic memory. However, a minute or two of recall performance only yields a couple dozen responses or so, which is a small number of samples for discriminating among candidate statistical models. Rhodes and Turvey (2007) were interested in determining IRI distributions in category recall tasks, so they asked participants to name animals for 10–20 min, which generated over 100 responses per participant, on average. The authors found IRI distributions for all subjects to resemble an inverse power law, P(IRI) ~1/IRI^α^, where α ~ 2 using logarithmic binning methods. This finding is intriguing because many studies of animal foraging, which may be analogous to memory foraging in category recall, have found a very similar inverse power law relationship (Viswanathan et al., 1996; Sims et al., 2008).

The inverse power law in IRI distributions suggests that semantic memory may be clustered across multiple scales. Memory searches may reflect this clustering, and in doing so, yield inverse power laws in path lengths. The hypothesized connection between semantic memory structure and IRI distributions raises two issues to be addressed. First, estimating inverse power law distributions from empirical data is an ongoing debate in many areas of cognitive science, and science writ large (Edwards et al., 2007; Clauset et al., 2009). Inverse power laws are difficult to distinguish from alternate functions like truncated power laws and lognormal distributions. The problem is that alternate functions are distinguished by very small numbers of rare, extreme observations. It is difficult to estimate their probability from limited amounts of data, and estimates can be biased by measurement conditions that restrict the range of possible observations. These issues aside, data have been mounting from more and more studies, under wider ranges of measurement conditions. The emerging picture is that behavior closely follows power law distributions under a wide range of conditions, albeit more like truncated power laws under restrictive measurement conditions (Kello et al., 2010; Rhee et al., 2011).

The second issue is that power law distributions in search paths could reflect random search strategies as simple, efficient heuristics. In particular, Viswanathan et al. (1999) showed that *Lévy walks* cover a search space with maximal efficiency when α = 2, under certain simplifying assumptions. Lévy walks can be expressed as path segments with randomly sampled directions, and lengths sampled from an inverse power law distribution, P(L) ~1/L^α^, where 1 < α < 3. The original analysis was restricted to sparse, randomly distributed targets that could be replenished after being foraged (Viswanathan et al., 1999). The analysis has been generalized since then to clustered distributions and non-replenishing targets which interestingly produced increased performance when the model was given some level of memory (Ferreira et al., 2012). Lévy walks are random and memoryless, however, other search strategies may take advantage of spatial correlations created by clustering, such as alternating search modes (Benhamou, 2007) or Bayesian search algorithms (Cain et al., 2012). Such strategies may also produce Lévy-like search trajectories (Boyer et al., 2006), even if they do not explicitly implement a Lévy walk.

Here we hypothesize that IRI distributions in category recall do reflect the structure of semantic memory. Our hypothesis is motivated by studies that have expressed semantic memory in terms of *scale-free networks* (Albert and Barabási, 2002), i.e., networks with power law degree distributions. In one study, Steyvers and Tenenbaum (2005) used data from both online and offline measures of semantic associations to construct semantic networks, where nodes were words and concepts, and links were associations among them. Online measures were collected from databases of semantic association norms, and offline measures came from Roget's thesaurus and Miller's encyclopedic WordNet database (Roget, 1911; Miller George, 1995). Clustering in the resulting networks was reflected in their *degree distribution*, i.e., the number of connections a given node has to other nodes, where each node is a word and connections reflect semantic associations/similarities. Specifically, node degrees were found to be power law distributed, where the probability of observing a node with degree *d* was inversely related to *d*, i.e., P(*d*) ~ 1/*d*^β^, where β ~ 1.8 for the directed association network and ~3 for the other networks.

In another study by Masucci et al. (2011), Wikipedia pages were treated as the nodes of a semantic network, where connections among pages were based on similarities in the probability distributions of content words used on pages. Again a power law distribution was found, P(*d*) ~1/*d*^β^, where β ~ 2.4. Another study that constructed a language network based on co-occurences of words in sentences (Cancho and Solé, 2001) also found a power law distribution P(*d*) ~1/*d*^β^, where β ~ 2.6. In more recent study, Morais et al. (2013) used a “snowballing” technique to analyze semantic networks created from individual participant data, instead of aggregate data. They found power law-like distributions that were truncated by an exponential cut-off for the most rare, extreme observations.

The goal of our study was to test whether power law-like IRI distributions and other findings from semantic category recall experiments could be explained by search over a semantic scale-free network. Previous work explained patch-like patterns in category recall in terms of random walks over semantic networks (Abbott et al., 2012), and we similarly aim to explain these patterns, but with an emphasis on the scale-free nature of semantic networks. We tested our hypothesis by (1) building a semantic network for animals based on Wikipedia; (2) determining whether the network is scale-free; (3) simulating random walks with memory over the structured Wikipedia network; (4) comparing these simulations with a scrambled control network; and (5) comparing simulated recall performance with observed recall data reported recently (Thompson et al., 2013). If the Wikipedia model simulates observed data better than the scrambled control, then we have evidence that Lévy-like patterns may arise from simple search processes ranging over scale-free search spaces. In the end, we discuss these processes and hypothesized reasons for scale-free structures in memory and semantics.

## Materials and methods

### Semantic networks for animals

While there are many ways to build semantic networks from empirical data, we chose to use Wikipedia because of its size, and because its entries explicitly and unambiguously representing the encyclopedic meanings of concepts. However, to test whether our semantic network is idiosyncratic to Wikipedia, we compared it with a second network built the same way, but using statistical co-occurrence data instead. In particular, we used the set of animals from Hills et al. (2012), and built a network using measures of semantic similarity derived from BEAGLE (Jones and Mewhort, 2007).

Wikipedia can be thought of as a vast, global repository of semantic information. As such, a number of methods have been developed to use Wikipedia for semantic processing and representations. Most of these methods focus on using Wikipedia to estimate the semantic relatedness of words or texts. Perhaps the most well-known method is Explicit Semantic Analysis (Gabrilovich and Markovitch, 2007), which uses Wikipedia pages as the dimensions for a semantic space. A given word or text is represented as a weighted vector over these dimensions, where each weight is set according to the word's frequency of occurrence in the corresponding Wikipedia page. ESA can be thought of as a variant of latent semantic analysis (Dumais and Landauer, 1997), with the advantage that the dimensions of co-occurrence vectors are explicitly semantic, given that Wikipedia pages are encyclopedic entries. In support of this advantage, ESA vector similarities have been shown to correlate strongly with human judgments of similarity ratings. Other Wikipedia-based methods have used the words in Wikipedia page titles (Strube and Ponzetto, 2006), or the hyperlinks among Wikipedia pages (Milne and Witten, 2008), as bases for measures of semantic relatedness.

While semantic relatedness is important for our purposes, our main goal is to define a semantic network. As mentioned earlier, Masucci et al. (2011) built a semantic network by connecting Wikipedia pages as nodes. Connections were based not on hyperlinks among pages, but rather, on each page's individual probability distributions over words. That is, the authors extracted a core set of content words for each Wikipedia page, excluding all function words and stripping words of their derivational inflections (i.e., words were lemmatized). The frequencies of resulting word lemmas were normalized to create a probability distribution for each Wikipedia page. Network connections between pages were created on the basis of overlap in probability distributions, as measured by their Jensen-Shannon Divergence (JSD),

$$\text{JSD}\left(P∥Q\right)=\frac{1}{2}D\left(P∥M\right)+\frac{1}{2}D\left(Q∥M\right)\text{where }M=\frac{1}{2}\left(P+Q\right)$$

Where *P* is a probability distribution, and *D*() is the Kullback-Liebler divergence:

$$D\left(P∥Q\right)=\sum_{i}\ln\left(\frac{P\left(i\right)}{Q\left(i\right)}\right)P(i)$$

JSD is an information theoretic divergence measure that calculates the relative informational entropy between two distributions. It is symmetric and normalized between 0 and 1 for maximal and minimal overlap. Unlike ESA, it is a direct measure of how related two Wikipedia pages are, with respect to their relative frequencies of word usage in pairs of articles. Masucci et al. (2011) created a semantic network by linking any two pages with a JSD below a given threshold. The threshold was chosen to be just lenient enough such that every page was interconnected by a single network (i.e., the minimum spanning tree). The resulting network was scale-free.

For the present study, we replicated the method used by Masucci et al. (2011) to create a semantic network for just the Wikipedia pages on animals. Animal Wiki pages were found using the Dbpedia ontology (Auer et al., 2007) which contains a list of all pages in Wikipedia associated with a given tag. A list of 5701 pages tagged as “animal” was compiled, after excluding all stub articles, redirect pages and articles with less than 500 words of main text. Formatting, references, and function words were removed from these pages, leaving only the article text describing the given animal. Using the Python natural language toolkit, inflectional variations were removed to create a probability distribution over lemmas for each animal page.

**Figure 1:**
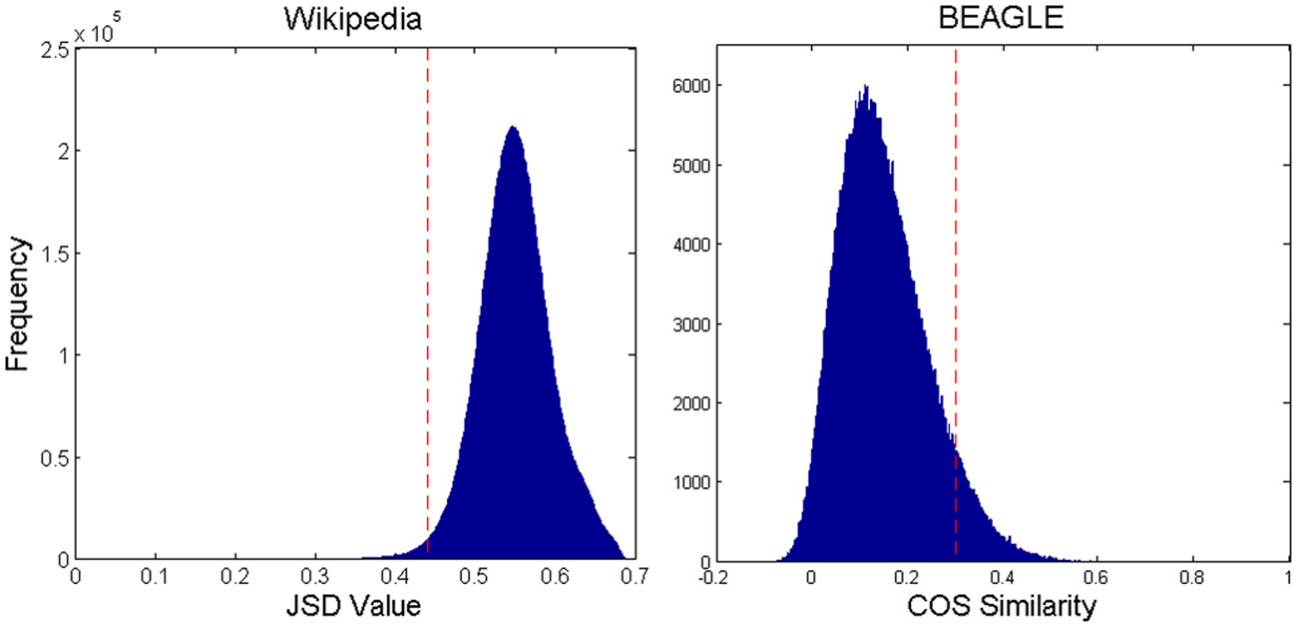
**Histogram of all pairwise JSD values for Wikipedia and BEAGLE animal pages, with the network connection threshold shown by the dashed vertical line.** For Wikipedia, smaller values mean greater similarity. For BEAGLE, it is the reverse.

JSDs were calculated for all pairs of animal pages, and a histogram of these as well as all cosign similarity values from BEAGLE is shown in Figure 1. We used an iterative process to find the similarity threshold that would keep only enough connections (excluding self-connections) to make a single network for 90% of the animal pages of either set. We discarded 10% from the Wikipedia network because most of these pages turned out to be lists of technical terms relating to the species, or other such anomalous entries that were nomadic with respect to JSDs. The same was done for the BEAGLE set of animals. As can be seen in Figure 1, the final threshold values for Wikipedia (0.44) and (0.30) for BEAGLE retained only the one-sided tails of Gaussian-shaped histogram of similarity values. Thus, connections are made only for highly similar pages relative to the distribution. The final network was formed by connecting all page nodes with pairwise similarities more similar than the set threshold and discarding all connections with less similarity. Finally, we constructed a scrambled control network with the same number of nodes and connections as the Wikipedia network, but with connections assigned at random (avoiding duplicates).

**Figure 2:**
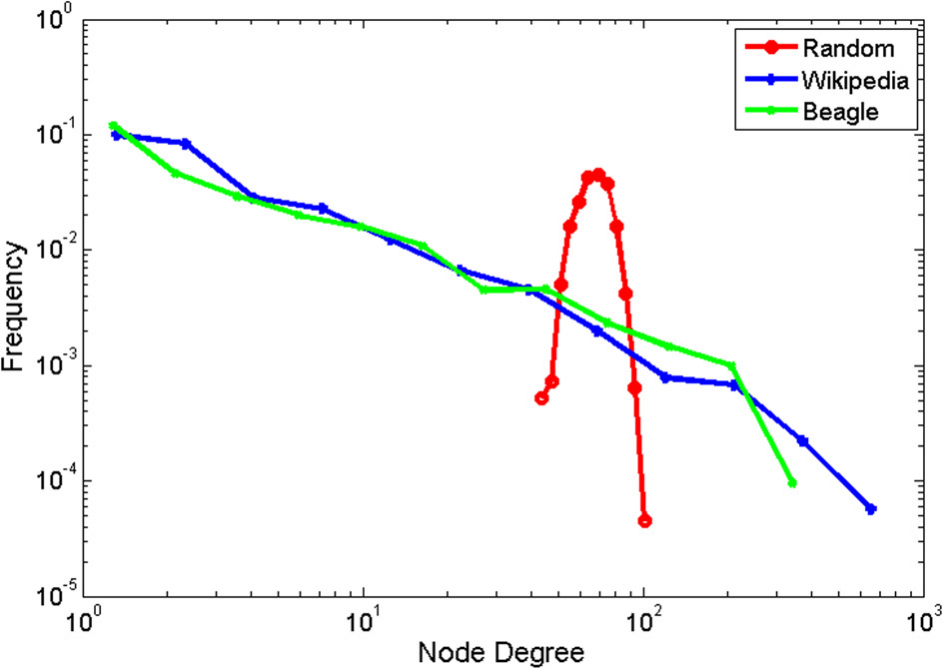
**Probability distributions for node degrees for the Wikipedia animal network, Beagle animal network, and scrambled control network.** Data are binned logarithmically, and shown in double logarithmic coordinates.

The resulting degree distributions are shown in log-log coordinates in Figure 2. The Wikipedia and BEAGLE networks exhibited remarkably similar same scale-free distribution, as seen in the same negative linear relationship in logarithmic coordinates. By contrast, the parabolic shape for the random network indicated a lognormal-shaped distribution with *M* = 68.29 and *SD* = 8.22. Mean and variance are measures of characteristic scale, whereas scale-free distributions, like those observed for Wikipedia and BEAGLE, have no characteristic scale. A better parameterization of scale-free distributions is the exponent of the power law relationship, which can be estimated by the negative of the slope of a regression line (with logarithmic binning; Newman, 2005). The slope was 1.14 for the Wikipedia network is 1.14, and 1.31 for the BEAGLE network. These slopes were shallower than that found by Masucci et al. (2011) for the whole of Wikipedia, but within the typically observed range for scale-free networks.

### Network search model

The network models presented thus far are simple, static structures in need of dynamics to simulate memory and semantic processes. Perhaps the most well-known dynamic used in semantic and language network models is spreading activation (Collins and Loftus, 1975). The construct of spreading activation is flexible enough to adapt for simulating numerous aspects of processing, but this flexibility comes at the cost of numerous free parameters that need to be set. Here we are focused on the category recall task, which can be operationalized more simply as a random walk over semantic memory. That is, category members can be recalled in any particular order, guided only by the general principle that successive retrievals tend to be semantically related. A walker process hops from one node to another via network connections based on semantic relations, and each node may or may not be retrieved on each hop. The only free parameters concern whether retrieval occurs or not at each node, and the temporal duration of each hop.

We implemented memory for prior retrievals in the model by simply marking each node as retrieved or not, and the probability of retrieval upon visiting a node was 0% if marked as retrieved (i.e., participants rarely repeat a previously retrieved item), and 10% if not. This stochastic component was one source of randomness, and the other source was direction of hopping, which was chosen at random from each node's set of connections. Varying the stochastic parameter had little effect on the pattern of results (see Figures S1–S4), so we report just one representative example at 10%. For the temporal duration of each hop, there are multiple mechanisms by which we could arrive at a measure of duration that make different assumptions about the processes underlying memory search. Two measures are reported for the distribution of IRIs. The first measure calculates the time between item retrievals as a summation of the number of intervening hops. The second measure calculates the time between retrievals as the sum of degrees (numbers of connections) for each node that is hopped. These walk processes simulate IRIs as being influenced by two main factors. One is the proportion of unretrieved items in the neighborhood of a current node, and the other is the overhead in choosing among connections. The hop measure is a relatively stronger test of the model, because counting hops does not directly reflect the scale-free structure of the Wikipedia network.

## Results

Each random walk was initiated at a randomly chosen node in the network, and ran until 400 animal nodes were retrieved. Simulated IRIs were compared with results from a recently reported category recall experiment (Thompson et al., 2013). Nineteen participants recalled items for 20 min to produce an average of 117 (*SD* = 38.6) animal names per session. A random walk was initiated 20 times each for the Wikipedia animal network and scrambled control network. Walks were run to retrieve more items than in the experiment, on the assumptions that the typical college student has significantly less than ~5000 animal names available for recall and that the number of responses are proportional to the number of items available for retrieval. Thus, longer simulation runs were meant to roughly normalize the proportion of items recalled.

Sequences of animal name responses are shown in Table 1 for an example participant and example runs from the scale-free animal network and scrambled control network. One can see the clustering of items that is typically found in category recall experiments, and a similar clustering for the animal network, albeit simulations tended to retrieve less common animal names simply because Wikipedia has so many uncommon animal entries. As one would expect, no such clustering is evident for the scrambled control network.

**Table 1:** **Example series of animals recalled by a participant and network walker model runs over Wikipedia animal network and scrambled control network**.

**Participant**	**Wikipedia**	**Random**
Seals	Lovebird	Donkey
Shrimp	Indian Peafowl	Kingfisher
Sea Lions	Peregrine Falcon	Bothrops alternatus
Elephant Seals	Barn Owl	Stock Dove
Blue Whale	Great Frigatebird	Giant isopod
Sperm Whale	Jamaican fruit bat	Archerfish
Orca	Mexican Free-tailed Bat	Ctenophora

**Figure 3:**
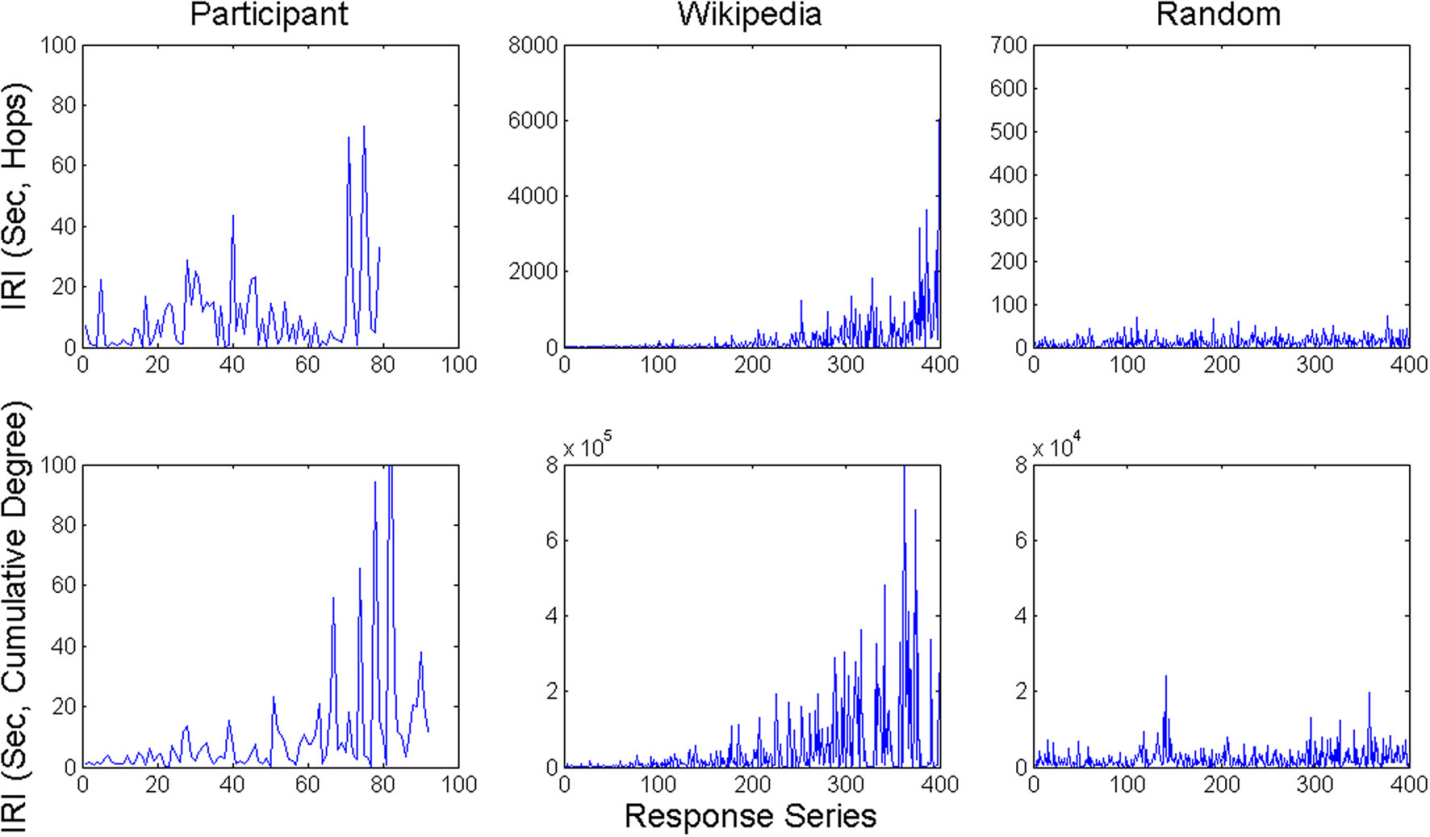
**Example time series of IRI's from a participant in the category generation task and network walker model runs over both the Wikipedia animal network and scrambled control network**.

IRI sequences are shown in Figure 3 for two example participants, and example runs from the animal and scrambled control networks using both response time measures. For the experiment and animal network, one can see mostly short IRIs, relatively speaking, interspersed with a small number of substantially longer IRIs, plus a few particularly long ones. Moreover, longer IRIs have a tendency to appear later in recall sequences. This tendency arises in the model because unretrieved items become scarcer over time, and by hypothesis, this tendency arose in the experiment for the same reason. Unlike the experiment and animal network, the scrambled control network does not exhibit such long IRIs relative to the mean. This difference held for both the hop and cumulative degree measures.

**Figure 4:**
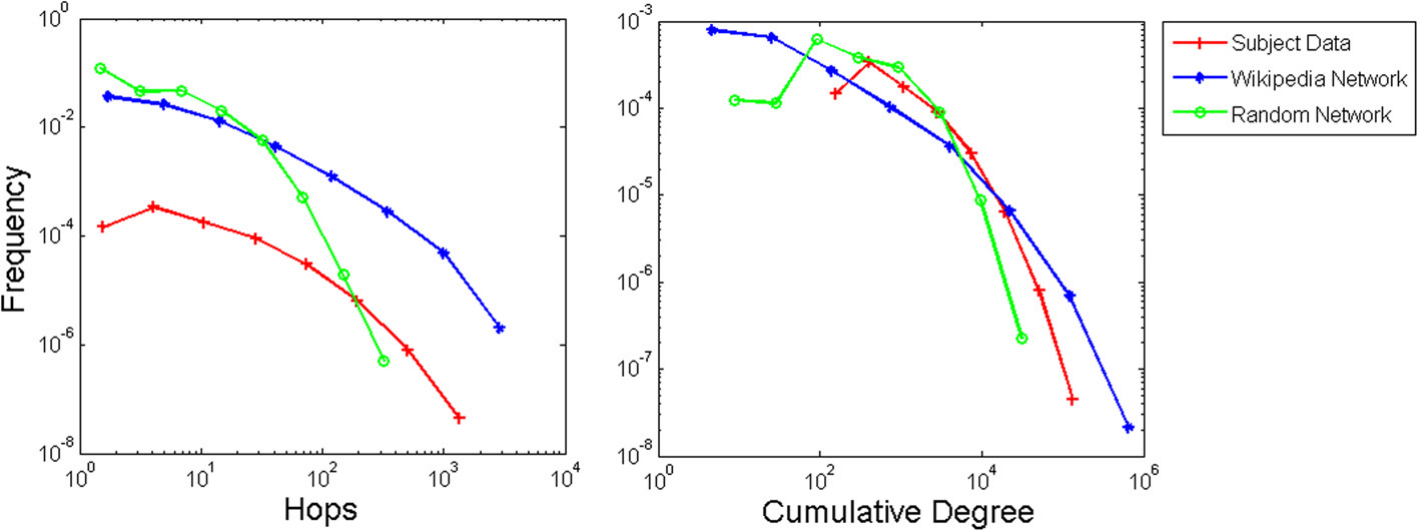
**IRI distributions in log-log coordinates (using logarithmic binning) for the experiment, Wikipedia animal network, and scrambled control network, aggregated over participants and simulation runs, respectively**.

Distributions of IRIs are shown in Figure 4 for experiment and model data using both model IRI measures, with participant data scaled to match the corresponding measure. Both the hop and cumulative degree measures result in power law-like distributions that resemble the participant data. The scrambled control network produced a more truncated, exponential-like distribution. We used multi-model inference (Burnham and Anderson, 2002; Edwards et al., 2007; Sims et al., 2008) to test the function that best fit the data. For the experiment, four participants were best fit by the Pareto inverse power law, and the remaining participants were best fit by a lognormal distribution (akin to a constrained or truncated power law in this case). Using the hop measure, all 20 runs were best fit by a lognormal distribution for the Wikipedia network, whereas all 20 runs were best fit by an exponential distribution for the random network. Using the cumulative degree measure all 20 runs were best fit by a lognormal distribution for the animal network and 16 runs were best fit by a lognormal distribution for the random network with 4 runs best fit by an exponential (full table of model fits included in Supplementary Materials). Thus, the hop measure is clearest in showing the distinction between Wikipedia and random networks.

Thus, far, the simulations show that power law-like IRI distributions in category recall experiments (i.e., truncated power laws that sometimes appear as lognormal) may arise from search processes that walk over scale-free semantic networks. The implication is that Lévy-like patterns in category recall data may arise from the structure of the search space, rather than the intrinsic properties of a random Lévy walk. Another source of evidence is the clustering of retrievals, as shown qualitatively in Table 1. A search process that reflects clustering in the structure of semantic space should naturally produce clustering in its retrievals.

**Figure 5:**
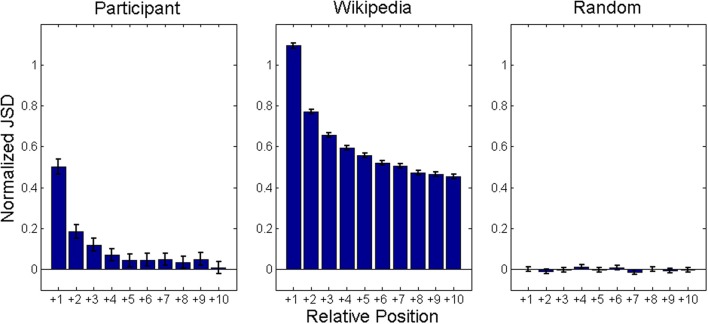
**Mean normalized JSD values between animal names produced 1–10 responses apart in category recall sequences.** JSD values normalized by mean and standard deviation all possible JSD values between items produced. Analysis based on Figure 1 from Hills et al. (2012).

To test whether the Wikipedia model also exhibits clustering in its retrievals, we examined the relationship between semantic similarity and positional distance in recall sequences. Consistent with its scale-free clustering, the Wikipedia model should show a general gradient of diminishing semantic similarity between two items as the number of intervening retrievals increases. If the human data contains a similar pattern, we will have further evidence for search processes operating over scale-free networks. Mean semantic similarity, as measured by normalized JSDs (Z scores), is shown in Figure 5 as function of number of intervening items in sequences. The graphs show a clear similarity gradient for the experiment and animal network, but not the scrambled control. Our simulated walk process accounts for the observed gradient because connections are based on a measure of semantic similarity. The walker tends to retrieve next items similar to current ones because usually it does not need to traverse many connections until it finds an unretrieved node and successfully retrieves it. When applied to the scrambled control network, the walker no longer exhibits this gradient because connections are not based on semantic similarity.

**Figure 6:**
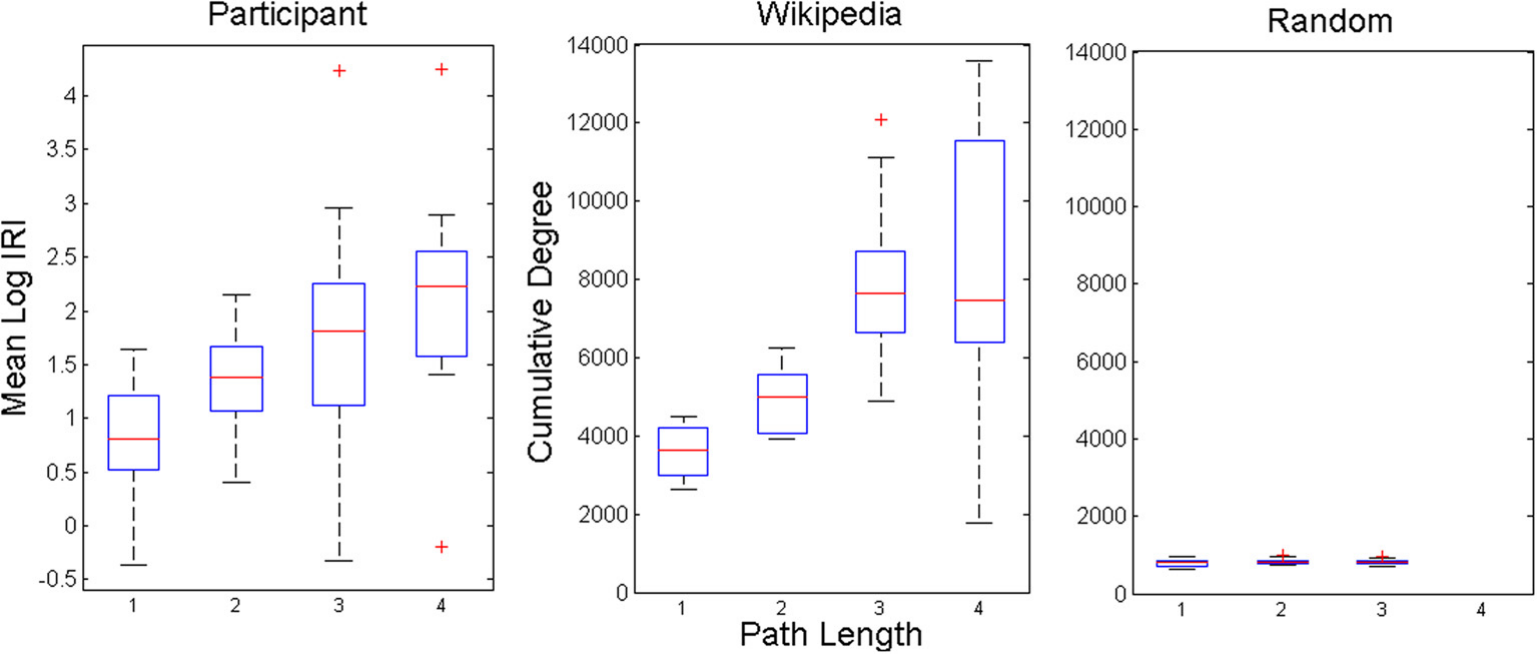
**Averaged response time measures between items with intervening network path lengths of 1–4.** Box plots show median and quartile values with outlying values (past 1.5 IQR) represented as pluses.

Our final test of the model is to examine the relationship between IRIs and network structure. A random walk between two given nodes of a network should require more hops as the distance between those nodes increases. A common metric of distance on networks is minimal path length, that is, the fewest number of hops needed to travel between two given nodes. Minimal path lengths were computed for all pairs of nodes corresponding to observed IRIs in the experiment, and the two model conditions. Lengths ranged between one and six for the experimental data, between one and seven for the animal network, and one and three for the scrambled control network. However, only lengths up to four were analyzed because <1% of the experimental data and 3.9% of the animal network data contained lengths greater than four. Mean IRIs are plotted as a function of minimal path length in Figure 6. IRIs were logarithmically transformed to account for their power law-like distributions. There was a significant effect of minimal path lengths on IRIs for the experimental data at the *p* < 0.01 level, *F*_(3, 66)_ = 9.06, *p* < 0.001, as well as the animal network data, *F*_(3, 76)_ = 24.96, *p* < 0.001. The network showed this relationship to weaken at length 4, relative to the empirical data, presumably because of compounding effects of noise. For the scrambled control network, there was no reliable relationship between IRIs and path length, *F*_(2, 57)_ = 1.17, *p* > 0.3. These results show that search dynamics in both the experiment and model reflected the structure of semantic space, as measured by distance between nodes in a semantic network.

## Discussion

Our results show that category recall performance can be simulated as a random walk process with memory over a scale-free semantic network. Clustering of animal name responses was shown to arise from clustering in inherent in scale-free network spaces, for both Wikipedia and BEAGLE networks. Our model explained IRIs as a function of traversing scale-free semantic spaces, rather than a stochastic component of dynamics that stipulates power law variations. Scale-free network structure accounted for IRIs between particular animal names in terms of minimal path lengths, as well as IRI distributions in terms of heavy-tailed functions. Observed and model distributions were highly similar to each other, and very similar to inverse power laws, albeit more limited versions of power laws that more closely resembled lognormal functions.

The effect of clustering in the Wikipedia network is generally consistent with theories that emphasize search over “patches” of items in memory (Troyer et al., 1997). The classic cluster-switching hypothesis holds that people alternate between producing words within subcategories and switching between these categories, two distinct search dynamics. By contrast, the Wikipedia model implements one search dynamic with two inherent timescales: Node traversal on a fast timescale, and foraging memory on a much slower timescale. That is, the walker algorithm becomes affected by its traversal history over time because the model remembers where it has been. This produces the appearance of distinct category switches when particularly many node traversals are required to find a new memory item. However, in our model there is only truly one mode of search and there is no characteristic scale to transitions just a certain percentage of transitions which will be distinctly large. Abbott et al. (2012) showed that similar walker dynamics can account for results previously interpreted as evidence for patch foraging. It remains to be seen whether future studies will reveal discriminating evidence between these different theories of search, or whether their commonalities will lead to a theory that integrates aspects of scale-free structure and patch-like switching dynamics.

Toward this end, future studies may investigate different methods of theorizing and modeling semantic spaces, and search dynamics over them. Our network structure and walker dynamics were formalized to be as simple as possible, given the data to be explained. Nonetheless, the model required short-term memory to avoid recalling the same items repeatedly, and the number of connections per node (i.e., neighborhood size) was optionally added as a factor that may affect IRIs. These additions are supported by previous studies on inhibition of return (Klein, 2000) and neighborhood effects (Vitevitch and Luce, 1998), but further work is needed to investigate how our modeling approach may be expanded to explain a broader range of memory phenomena. As mentioned earlier, spreading activation dynamics may provide a more versatile class of dynamics, or multiple random walk processes executed in parallel, with or without interactions among them. With respect to our model of semantic space, it would be informative to investigate and compare how other measures of semantics (e.g., LSA, ESA, WordNet, etc.) and other forms of representation (e.g., vector spaces, holographic spaces) might be used to theorize memory foraging and account for category recall data.

Finally, memory foraging is just one search domain in cognitive science. Information search through the web and other human-computer interfaces may also be usefully theorized as foraging (Pirolli and Card, 1999), and there is already evidence that visual search exhibits Lévy-like eye movement trajectories (Stephen and Mirman, 2010; Rhodes et al., 2011). Moreover, studies have found power law structures in how information is distributed over the internet (Faloutsos et al., 1999), and how visual features are distributed in the visual world (Field, 1987). The present study suggests that Lévy-like behaviors may be a general consequence of search over scale-free spaces.

## Conflict of interest statement

The authors declare that the research was conducted in the absence of any commercial or financial relationships that could be construed as a potential conflict of interest.
