# Supplementary material for: Walking across Wikipedia: a scale-free network model of semantic memory retrieval
Source: Front Psychol. 2014 Feb 11;5:86. doi: 10.3389/fpsyg.2014.00086 (PMC3920107; doi:10.3389/fpsyg.2014.00086)
Supplement: Supplementary file 1 [file DataSheet1.PDF]

Figures 1-4: Different IRI Measures and Values of Stochastic Parameter

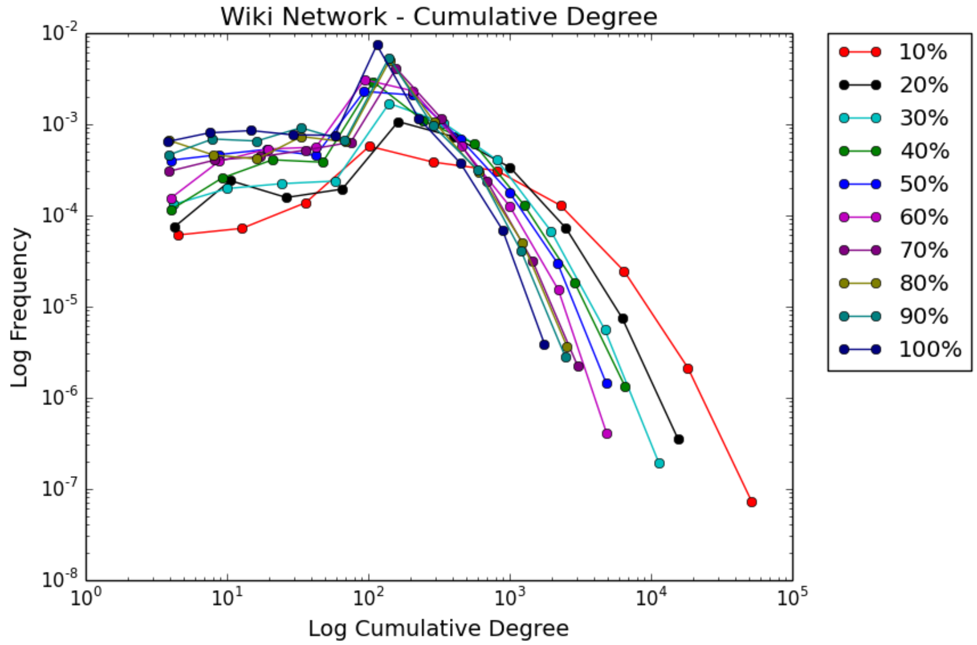

Figure 1: IRI distributions in log-log coordinates (using logarithmic binning) for the Wikipedia animal network aggregated over simulation runs, using cumulative degree of nodes hopped to as the IRI measure. Stochastic parameter varied between 10% and 100%

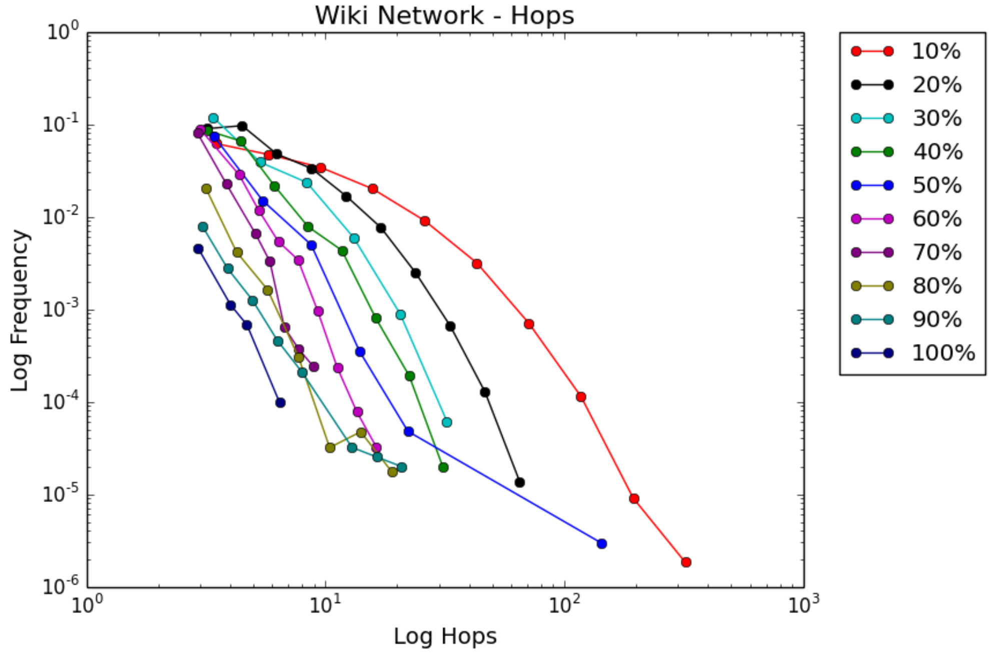

Figure 2: IRI distributions in log-log coordinates (using logarithmic binning) for the Wikipedia animal network aggregated over simulation runs, using number of hops between retrieved items as the IRI measure. Stochastic parameter varied between 10% and 100%

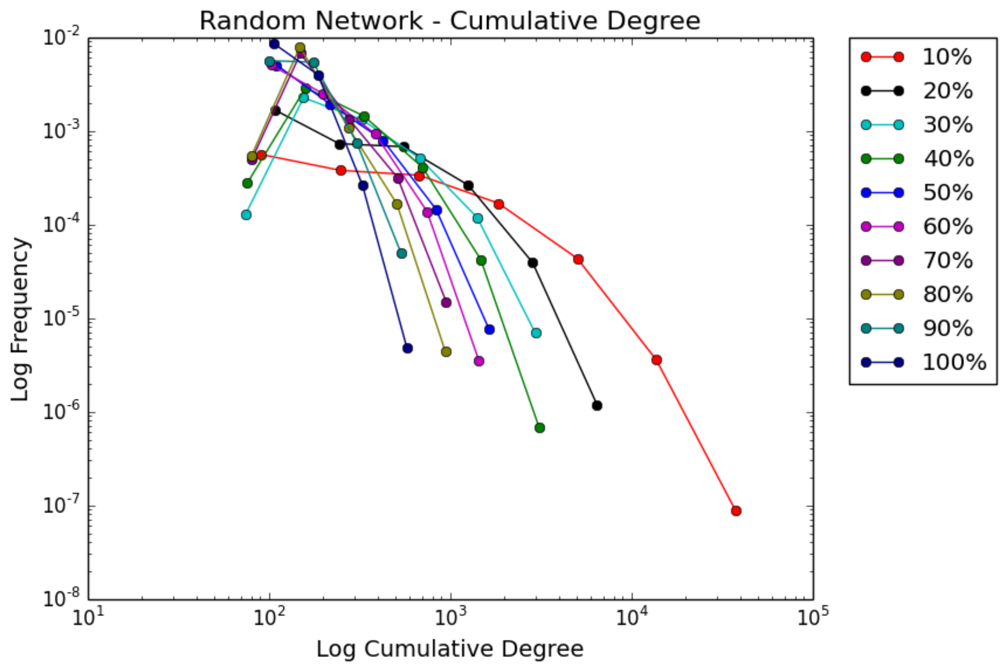

Figure 3: IRI distributions in log-log coordinates (using logarithmic binning) for the scrambled random network aggregated over simulation runs, using cumulative degree of nodes hopped to as the IRI measure. Stochastic parameter varied between 10% and 100%

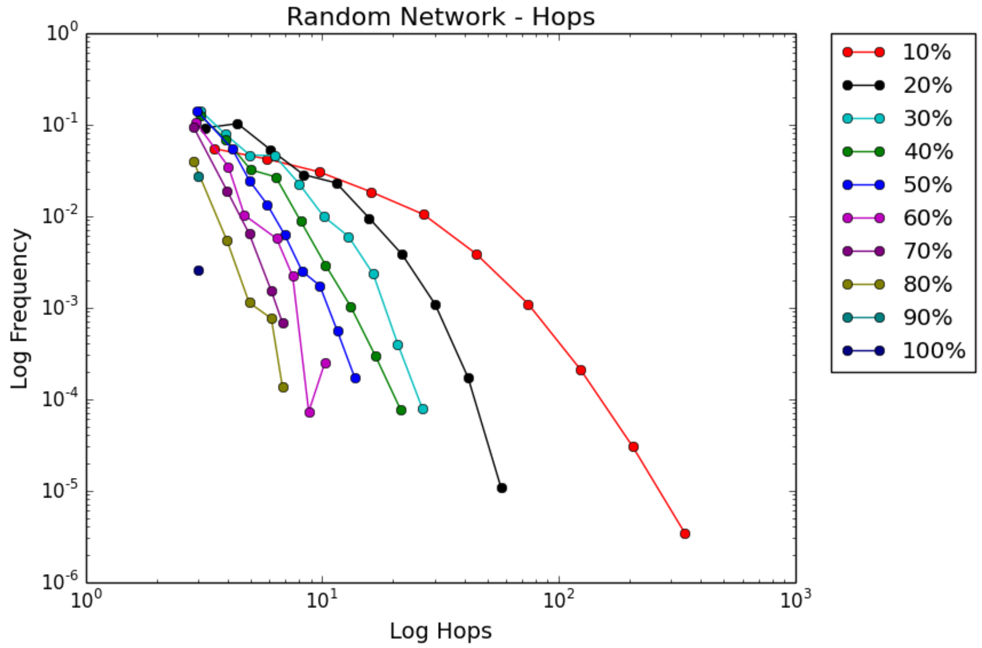

Figure 4: IRI distributions in log-log coordinates (using logarithmic binning) for the scrambled random network aggregated over simulation runs, using number of hops between retrieved items as the IRI measure. Stochastic parameter varied between 10% and 100%

Tables 1-5: Akaike Information Criterion Fits for Participant and Model Distributions

AIC Fits for Participant Data

Models

Gaussian	Lognormal	Exponential	Pareto
468.6782	339.9892	367.8493	242.6781
468.6782	339.9892	367.8493	242.6781
780.5172	302.8863	405.973	325.9817
421.7539	339.0311	394.3395	226.3423
863.2009	505.0102	584.2005	465.2123
355.4295	270.5866	287.2646	236.1523
355.4295	270.5866	287.2646	236.1523
599.9525	310.7494	375.3857	271.586
579.6425	359.3602	405.0317	324.4671
579.6425	359.3602	405.0317	324.4671
643.4927	464.6633	497.5717	356.5985
565.1196	341.8702	382.0506	369.8766
380.8543	309.4871	382.2825	372.5232
916.977	746.1884	862.274	752.9406
463.6083	346.1204	424.3585	406.2928
635.1966	450.7202	485.085	437.1692
289.7297	200.3311	218.7632	150.4919
838.5681	479.8574	555.7785	447.198
680.7908	441.7701	496.7641	344.5557

Table 1: Akaike information criterion fits of the four distributions tested for all 19 participants in the animal naming task. A smaller value indicates a closer fit.

AIC Fits for Wikipedia Network - Hops

Models

Gaussian	Lognormal	Exponential	Pareto
5833.5993	4378.6828	4816.8299	6540.789
5934.3763	4534.0287	4949.9285	6754.6374
5736.4691	4417.0854	4723.7271	6691.9652
5437.2394	4227.8643	4530.4839	6491.5427
5544.7251	4369.0069	4681.4494	6610.5194
5691.766	4542.1095	4830.7435	6742.514
5807.4093	4501.3766	4850.6444	6752.9725
5667.7862	4368.2001	4728.3651	6610.279
5864.841	4453.0493	4896.2513	6677.499
5790.0986	4418.6915	4830.4184	6619.5724
5311.003	4273.7916	4479.5491	6569.5823
5574.8911	4384.4815	4666.8719	6659.8401
5234.9872	4244.4785	4427.4126	6501.9122
5360.1777	4166.2238	4464.4036	6448.5639
5359.5205	4223.8391	4498.0667	6451.9413
5801.2513	4453.3413	4806.9269	6777.4158
5417.3037	4201.876	4479.0448	6468.8385
5978.982	4480.1875	4901.9803	6699.1861
5846.7585	4627.8907	4935.45	6908.6592
5689.6407	4423.3028	4737.0499	6690.5759

Table 2: Akaike information criterion fits of the four distributions tested for 20 runs of the network walker model using the Wikipedia network and the hop measure for IRI. A smaller value indicates a closer fit.

AIC Fits for Wikipedia Network - Cumulative Degree

Models

Gaussian	Lognormal	Exponential	Pareto
10390.2844	8834.0884	9366.6103	33649.2488
10463.7223	8976.4873	9483.0226	41332.1023
10292.4222	8846.2705	9256.1326	68980.043
10006.9006	8650.1184	9082.4709	39701.7854
10110.6961	8825.2892	9222.1676	27034.4783
10251.1145	8947.0448	9373.8202	47897.835
10353.5514	8928.6407	9381.4081	41194.4733
10226.3636	8821.4708	9277.2884	26950.9481
10397.9121	8849.7961	9431.9745	33527.2489
10345.2363	8851.7545	9380.7235	47252.5799
9873.3105	8681.813	9020.6638	26384.045
10139.2649	8787.0133	9204.1095	54109.1496
9807.8636	8703.7065	8973.1266	39968.0511
9865.619	8621.3859	8995.5569	26318.2596
9928.7183	8672.5272	9039.9844	33291.4053
10333.265	8882.5737	9329.0686	62390.9067
9962.5391	8619.3196	9000.846	19846.6759
10547.3018	8910.0957	9449.7025	27230.6413
10391.353	9069.8058	9474.9143	70519.1082
10255.0533	8899.2895	9296.4532	34208.1188

Table 3: Akaike information criterion fits of the four distributions tested for 20 runs of the network walker model using the Wikipedia network and the cumulative degree measure for IRI. A smaller value indicates a closer fit.

AIC Fits for Random Network - Hops

Models

Gaussian	Lognormal	Exponential	Pareto
3281.4075	3117.9899	2918.798	5605.5199
3197.1573	2925.7938	2758.4821	5360.8384
3127.2283	2943.0134	2757.6149	5368.2859
3771.2503	3048.0427	2917.378	5484.4392
3281.6594	2971.5854	2837.6403	5387.2477
3273.9017	2965.5947	2778.5109	5453.5271
3157.0249	2894.816	2734.9096	5346.7199
3165.8593	2953.2163	2773.2528	5452.6207
3398.8808	2994.201	2853.3439	5427.7273
3560.9986	2912.5942	2826.8913	5357.7708
3236.7924	2945.9622	2810.492	5345.686
3176.4683	2926.3593	2764.8704	5353.4342
3279.6732	2945.3465	2803.6404	5344.3114
3476.7841	3039.8847	2930.2076	5481.5723
3340.7819	2935.4811	2799.534	5384.1454
3210.0235	2979.7827	2802.0004	5449.1564
3217.5231	2998.5995	2801.8362	5487.8132
3368.4321	3034.2554	2888.4365	5447.9271
3200.4727	2984.5508	2795.4065	5417.1525
3116.2102	2917.5669	2736.1583	5370.1638

Table 4: Akaike information criterion fits of the four distributions tested for 20 runs of the network walker model using the random network and the hop measure for IRI. A smaller value indicates a closer fit.

AIC Fits for Random Network - Cumulative Degree

Models

Gaussian	Lognormal	Exponential	Pareto
7690.5722	7207.9133	7205.8745	85913.8205
7735.114	7081.7058	7131.2095	83935.9785
7443.574	6956.1595	6973.0214	156643.4624
7517.8013	7100.9541	7096.2987	148199.1011
7761.7343	7077.5508	7124.2133	38320.3856
7772.86	7006.956	7059.3738	359312.4638
7364.7051	6893.5146	6910.3223	555934.5342
7566.876	7012.5607	7041.3337	356819.297
7722.3902	7081.5851	7127.1398	55298.9333
7630.7227	6979.8732	7010.5228	165871.4855
7593.6443	7044.4451	7070.6777	242798.1458
7428.5899	6953.5826	6966.1478	43582.3349
7724.5304	7001.8146	7054.4319	561853.4224
7996.4958	7221.0168	7294.9049	62023.2043
7600.3474	7024.8327	7033.205	88430.9055
7676.7654	7014.7175	7053.3008	111061.9581
7678.1945	7033.0367	7070.7077	112412.1977
7855.1389	7172.9719	7207.0707	33174.6153
7337.8442	6924.1104	6930.3195	355445.245
7349.1829	6909.2475	6918.2684	234550.5102

Table 5: Akaike information criterion fits of the four distributions tested for 20 runs of the network walker model using the random network and the cumulative degree measure for IRI. A smaller value indicates a closer fit.
